# Electrochemical Evaluation of Tumor Development via Cellular Interface Supported CRISPR/Cas Trans-Cleavage

**DOI:** 10.34133/2022/9826484

**Published:** 2022-04-06

**Authors:** Liangfen Cheng, Fuhan Yang, Longfei Tang, Lelin Qian, Xu Chen, Feng Guan, Juan Zhang, Genxi Li

**Affiliations:** ^1^Center for Molecular Recognition and Biosensing, School of Life Sciences, Shanghai University, Shanghai 200444, China; ^2^Department of Urology, Shanghai Tenth People's Hospital, School of Medicine, Tongji University, Shanghai 200092, China; ^3^College of Life Science, Northwest University, Xi'an 710127, China; ^4^State Key Laboratory of Pharmaceutical Biotechnology and Collaborative Innovation Center of Chemistry for Life Sciences, Department of Biochemistry, Nanjing University, Nanjing 210093, China

## Abstract

Evaluating tumor development is of great importance for clinic treatment and therapy. It has been known that the amounts of sialic acids on tumor cell membrane surface are closely associated with the degree of cancerization of the cell. So, in this work, cellular interface supported CRISPR/Cas trans-cleavage has been explored for electrochemical simultaneous detection of two types of sialic acids, i.e., N-glycolylneuraminic acid (Neu5Gc) and N-acetylneuraminic acid (Neu5Ac). Specifically, PbS quantum dot-labeled DNA modified by Neu5Gc antibody is prepared to specifically recognize Neu5Gc on the cell surface, followed by the binding of Neu5Ac through our fabricated CdS quantum dot-labeled DNA modified by *Sambucus nigra* agglutinin. Subsequently, the activated Cas12a indiscriminately cleaves DNA, resulting in the release of PbS and CdS quantum dots, both of which can be simultaneously detected by anodic stripping voltammetry. Consequently, Neu5Gc and Neu5Ac on cell surface can be quantitatively analyzed with the lowest detection limits of 1.12 cells/mL and 1.25 cells/mL, respectively. Therefore, a ratiometric electrochemical method can be constructed for kinetic study of the expression and hydrolysis of Neu5Gc and Neu5Ac on cell surface, which can be further used as a tool to identify bladder cancer cells at different development stages. Our method to evaluate tumor development is simple and easy to be operated, so it can be potentially applied for the detection of tumor occurrence and development in the future.

## 1. Introduction

Evaluating tumor development is of great importance for clinic treatment and therapy. Currently, the optical visualization of stained biopsy specimens is still the main diagnostic method in clinic trial. However, such technique is invasive and easily leads to human error and misdiagnosis [1]. Meanwhile, intratumor heterogeneity brings the obstacle for the precise characterization of the developing stage of cancer [1, 2]. Although some other techniques, such as screening mammography [3], magnetic resonance imaging [4], and technicium-99 radiocolloid lymphatic mapping [5], have also been utilized to identify different stages of the corresponding cancer, these techniques have radiation damage to patients. And the high false-positive rate derived from these techniques make it a great job to explore more approaches for cancer diagnostics and tumor development evaluation.

Currently, tumor markers have been extensively explored for the diagnosis of cancers, because they are closely related with tumor stages, metastases, and tumor recurrence [6]. As one kind of tumor markers, sialic acid plays a key role in regulating cell adhesion, migration, and recognition between cells [7]. It is a common glycosylation form of glycoproteins. The change of glycosylation structure of glycoproteins on cell membrane surface is closely related to tumor development [8]. Cancer cells with nutritional deficiency can absorb sialic acid more, resulting in the occurrence of sialic acid on the cell surface [9]. The most common types of sialic acid are N-glycolylneuraminic acid (Neu5Gc) and N-acetylneuraminic acid (Neu5Ac) [10]. Neu5Gc is the hydroxylated form of Neu5Ac, catalyzed by cytosine monophosphate Neu5Gc synthetase (CMAH) [11]. In human body, the deletion of 92 bp fragment in exon results in the inactivation of CMAH [12]. In recent years, however, studies have revealed that Neu5Gc appears in human body in the form of exogenous [13], and it is closely related to a variety of cancers [14], such as colon cancer [15], bladder cancer [16], breast cancer, lung cancer, and ovarian cancer [17]. Under hypoxia, cancer cells can upregulate subunit B of respiratory complex II, i.e., an iron-sulfur [Fe2S2]-containing protein, making up for the lack of function of CMAH enzyme, and resulting in the high expression of Neu5Gc in cancer cells [18]. The appearance of Neu5Gc on the surface of cancer cell membrane makes tumor cells escape immune monitoring and provides opportunities for the malignant reproduction of tumor cells [19]. At the same time, Neu5Gc produces antibodies in the human body, resulting in occurrence of chronic inflammation which promotes the development of tumor [20]. Therefore, the amount of Neu5Gc and Neu5Ac is closely associated with the degree of cancerization of the tumor cell and tumor development.

CRISPR/Cas system is composed of clustered regularly interspaced short palindromic repeats (CRISPR) and CRISPR-associated proteins (Cas) [21, 22]. CRISPR/Cas system is a powerful gene editing tool [23], and it has been used in the other research fields. For instance, the diagnostic techniques developed by CRISPR/Cas may include specific high sensitivity enzymatic reporter unlocking, one-hour low-cost multipurpose and efficient system, and DNA endonuclease targeted CRISPR trans reporter [24]. On the other hand, Cas12a nuclease is composed of Cas protein and the corresponding crRNA. In the presence of target strand, crRNA and Cas protein can form a ternary complex, so as to activate the trans-cleavage activity and randomly cleave single-stranded DNA [25]. Because of its high specificity and efficient trans-cleavage activity, Cas12a can be used as a recognition element and amplification element.

In this work, the trans-cleavage ability of Cas12a on the surface of cell membrane has been investigated. Quantum dot-modified single-stranded DNA is designed to be linked onto cell membrane surface through specific recognition between sialic acids and antibody or lectin. The activated Cas12a can exhibit efficient trans-cleavage capability on the immobilized single-stranded DNA. Based on these designs and findings, a new method has been proposed to predict the degree of cell cancerization, serving for the evaluation of tumor development.

## 2. Results

### 2.1. Interface Supported CRISPR/Cas Trans-Cleavage for Dual Electrochemical Signal Output

**Figure 1:**
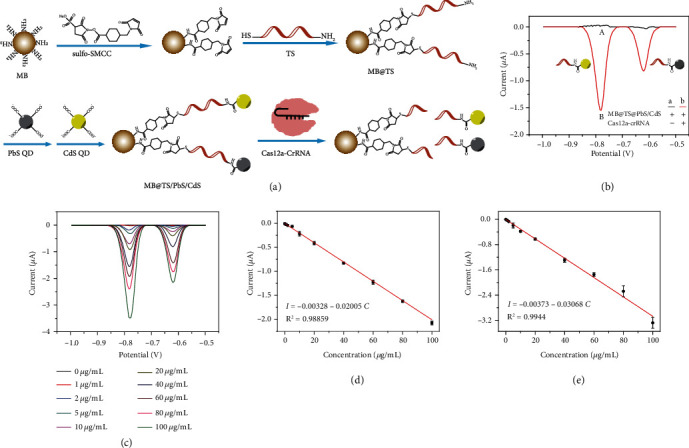
Interface supported CRISPR/Cas trans-cleavage for dual electrochemical signal output. (a) Schematic illustration for magnetic bead interface supported CRISPR/Cas trans-cleavage. (b) Anodic stripping voltammograms without or with Cas12a-crRNA. (c) Anodic stripping voltammograms obtained with different concentrations of MB@TS/PbS/CdS. Linear relationships between current values and amount of (d) PbS QDoligo and (e) Cds QDoligo cleaved by activated Cas12a on the surface of modified MB.

The principle of interface supported CRISPR/Cas trans-cleavage has been exhibited in Figure 1(a). Using sulfo-SMCC as a linker, TS can be modified onto the surface of magnetic bead to give MB@TS with the exposure of amino groups. Subsequently, the carboxylated PbS QD and CdS QD with molar ratio of 1 : 1 can react with MB@TS with the formation of amide bond, to obtain MB@TS/PbS/CdS. In the presence of Cas12a-crRNA, TS can be indiscriminately cleaved, resulting in the departure of QD-labeled oligonucleotide (QDoligo) from the surface of the modified magnetic bead.

Both PbS QD and CdS QD can simultaneously give the sensitive electrochemical peak at -0.62 V and -0.78 V by anodic stripping voltammetry after dissolving in nitric acid, respectively (Figure S1(a)) [26]. With the concentrations of PbS QD or CdS QD increasing from 0 ng/mL to 10000 ng/mL, the current values of the electrochemical peaks linearly decrease (Figure S1(b) and S1(c)). The linear equations of I = −0.00982–0.00304 C and I = −0.05238–0.00127 C, where I is peak current value and C is the concentration of QD, can be obtained for PbS QD and CdS QD, respectively. In the presence of Cas12a-crRNA, the similar electrochemical peaks at -0.62 V and -0.78 V can be observed for the supernatant solution after magnetic separation (Figure 1(b)), which can be separately attributed to PbS QDoligo and CdS QDoligo. The result well confirms that the Cas12a can be activated to cleave TS around the modified MB, leading to the release of QDoligo. In contrast, nearly no peak can be found in the absence of Cas12a-crRNA, due to no release of QDoligo. Different amounts of MB@TS/PbS/CdS have been further utilized to evaluate interface supported CRISPR/Cas trans-cleavage. With the increased amount of MB@TS/PbS/CdS, the increased amount of QDoligo can be released into the solution after cleaving by activated Cas12a. It can be found that the current values increase with the increasing MB@TS/PbS/CdS (Figure 1(c)). Moreover, good linear relationships can be separately obtained between the peak current values of PbS QDoligo or CdS QDoligo and the concentrations of MB. For PbS QD-modified MB, a linear equation of I = −0.00328 − 0.02005 C can be given (Figure 1(d)), and for CdS QD-modified MB, a linear equation of I = −0.00373 − 0.03068 C can be given (Figure 1(e)). These results signify that the interface supported CRISPR/Cas trans-cleavage can well mediate the output of electrochemical dual signals, which can be used to quantitatively analyze the amount of modified MB.

### 2.2. Cellular Interface Supported CRISPR/Cas Trans-Cleavage for Dual Electrochemical Signal Output

**Figure 2:**
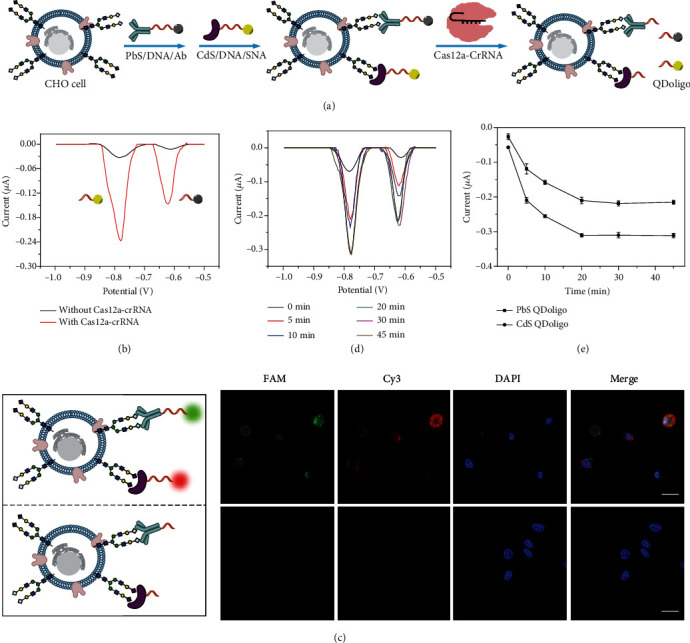
Cellular interface supported CRISPR/Cas trans-cleavage for dual electrochemical signal output. (a) Schematic illustration for cellular interface supported CRISPR/Cas trans-cleavage for electrochemical detection of Neu5Gc and Neu5Ac on CHO cell surface. (b) Anodic stripping voltammograms obtained with and without Cas12a-crRNA. (c) Laser confocal images of CHO cell labeled by FAM/DNA/Ab and Cy3/DNA/SNA (up) without and (down) with Cas12a-crRNA. Inset: schematic illustration for CHO cell without or with cleavage. Scale bar: 25 *μ*m. (d) Anodic stripping voltammograms obtained with different time for cell labeling by using PbS/DNA/Ab and CdS/DNA/SNA. (e) Current values versus different time for CRISPR/Cas trans-cleavage on the surface of cell.

Sialic acids on cell surface play key roles for cell adhesion, communication, and migration, and they have been considered as cancer markers in human body [27]. Herein, a new method to detect Neu5Gc and Neu5Ac on cell surface has been developed via dual electrochemical signal output based on cellular interface supported CRISPR/Cas trans-cleavage. The design of the new method has been illustrated in Figure 2(a). With the assistance of single-stranded DNA (Target strand, TS), Neu5Gc antibody and *Sambucus nigra* agglutinin (SNA) are separately labeled by PbS QD and CdS QD to give PbS/DNA/Ab and CdS/DNA/SNA, respectively. So, the sialic acids, Neu5Gc and Neu5Ac, can be specifically recognized by PbS/DNA/Ab and CdS/DNA/SNA, respectively. Subsequently, the activated Cas12a can indiscriminately and efficiently cleave single-stranded DNA (TS), resulting in the departure of QDoligo from the surface of cell. After centrifugation, the QDoligo in the supernatant solution can be detected by anodic stripping voltammetry after dissolving in nitric acid.

In our experiment, CHO cell, a Chinese hamster ovary cell which expresses Neu5Gc and Neu5Ac molecules [28], has been utilized. As shown in Figure 2(b), the addition of Cas12a-crRNA leads to the appearance of two obvious electrochemical peaks located at -0.62 V and -0.78 V, which can be attributed to the oxidation of PbS QD and CdS QD, respectively. It can be explained for the efficient cleavage of the activated Cas12a on single-stranded DNA around CHO cell. On the contrary, two small negligible peaks can be observed in the absence of Cas12a-crRNA, as a result of the occurrence of DNA around the cell. Moreover, FAM/DNA/Ab and Cy3/DNA/SNA have also been prepared for laser confocal imaging of CHO cell. As exhibited in Figure 2(c), the bright green and red fluorescence can be found without Cas12a-crRNA. In contrast, almost no green and red fluorescence can be observed after cleaving through the activated Cas12a. These results are in well agreement with those obtained through electrochemical method. In addition, single-stranded DNA on the surface of CHO cell can be entirely cleaved after 20 min (Figures 2(d) and 2(e)). Therefore, through cellular interface supported CRISPR/Cas trans-cleavage, our established electrochemical method can be applied to detect Neu5Gc and Neu5Ac on the cell surface.

### 2.3. Electrochemical Detection of Neu5Gc and Neu5Ac Based on Cellular Interface Supported CRISPR/Cas Trans-Cleavage

**Figure 3:**
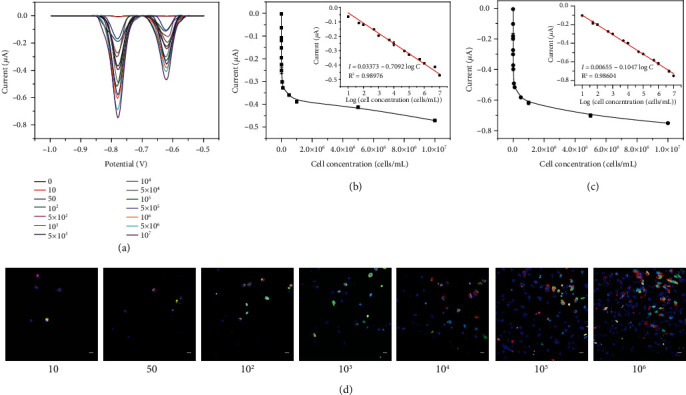
Electrochemical detection of Neu5Gc and Neu5Ac based on cellular interface supported CRISPR/Cas trans-cleavage. (a) Anodic stripping voltammogram with different cell numbers. (b) Linear relationship between the current values of electrochemical peak of PbS QDoligo and the number of cells. (c) Linear relationship between the current values of electrochemical peak of CdS QDoligo and the number of cells. (d) Laser confocal images of CHO cell with different amounts ranging from 10 to 10^6^. Scale bar: 25 *μ*m.

The established method in the work has been used to quantitatively analyze Neu5Gc and Neu5Ac on the surface of cell. As exhibited in Figure 3(a), along with the increased cell amount, the peak current values raise. It can be ascribed to the fact that the increased amount of PbS QDoligo and CdS QDoligo on the cell surface can be released into the solution due to the cleavage effect of the activated Cas12a on single-stranded DNA. Moreover, good linear relationships between the number of cells and the electrochemical signals of QDoligo can be found (Figures 3(b) and 3(c)). The linear equations of I = 0.03373 − 0.7092 LogC and I = 0.00655 − 0.1047 LogC, where I is peak current value and C is the concentration of cell, can be obtained for Neu5Gc and Neu5Ac, respectively. Meanwhile, for Neu5Gc and Neu5Ac, the lowest detection limit of 1.12 cells/mL and 1.25 cells/mL can be separately calculated through the interpolation of the mean plus three times the standard deviation of the zero standards. Different amounts of CHO cells have been imaged and analyzed by confocal microscopy. As shown in Figure 3(d), with the increase of the number of cells, the fluorescence becomes bright (Figure S3), indicating the consistency between electrochemical detection results and confocal imaging results. These results well signify that the established electrochemical method can quantitatively analyze the content of Neu5Gc and Neu5Ac on the cell surface.

### 2.4. Kinetic Study for Expression and Hydrolysis of Neu5Gc and Neu5Ac on Cell Surface through Ratiometric Electrochemical Method

**Figure 4:**
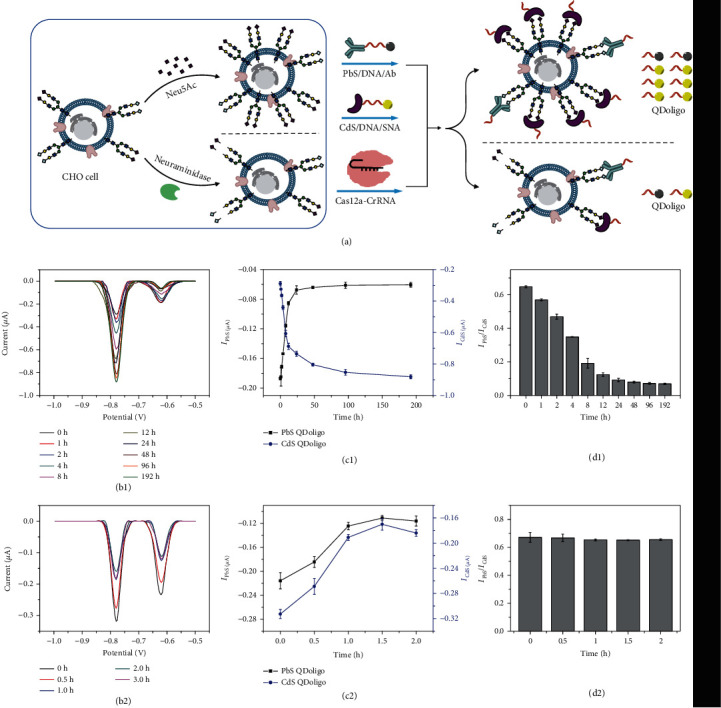
Kinetic study of expression and hydrolysis of Neu5Gc and Neu5Ac on cell surface. (a) Schematic illustration for expression and hydrolysis of Neu5Gc and Neu5Ac on cell surface. Anodic stripping voltammogram with different (b1) feeding and (b2) hydrolyzed time. Electrochemical peak current values of PbS QDoligo or CdS QDoligo versus different (c1) feeding and (c2) hydrolyzed time. The ratio of *I*_PbS_/*I*_CdS_ versus different (d1) feeding and (d2) hydrolyzed time.

The schematic illustration of detecting the kinetic changes of Neu5Gc and Neu5Ac molecules on the cell surface by the fabricated ratiometric electrochemical method is shown in Figure 4(a). Two obvious peaks attributed to the oxidation of CdS QD and PbS QD can be observed for CHO cells which is fed with Neu5Ac (Figure 4(b1)). The result confirms the simultaneous occurrence of Neu5Ac and Neu5Gc on the surface of CHO cells. Neu5Ac can be synthesized into CMP-Neu5Ac which can be hydroxylated into CMP-Neu5Gc in the cytosol, transported into the Golgi apparatus, and transferred to glycoconjugates by sialytransferases [29]. Along with the increase of feeding time, the peak current values of CdS QDoligo increase, whereas the values of PbS QDoligo decrease (Figures 4(c1)). On the one hand, the increased amount of Neu5Ac absorbed by CHO cell can transfer onto the surface of the cell, resulting in the increasing current values of CdS QDoligo. On the other hand, the corresponding increased CMP-Neu5Ac can completely transfer onto the surface of CHO cell through sialytransferases. It has been reported that the isolated Golgi apparatus utilizes CMP-Neu5Ac and CMP-Neu5Gc without significant preference [30]. Meanwhile, there is no significant preference for CMP-Neu5Gc or CMP-Neu5Ac of the porcine and bovine GalNAc *α* 2, 6-sialyltransferases purified from submandibular glands [31]. So, the increased transference of the large amount of CMP-Neu5Ac will undoubtedly inhibit the transference of CMP-Neu5Gc, resulting in the decrease of current values of PbS QDoligo along with the prolonged feeding time.

In addition, we have also established a kinetic model of neuraminidase hydrolysis. By acting neuraminidase on CHO cells, Neu5Gc and Neu5Ac molecules on the cell surface are hydrolyzed to make the cell surface carry different contents of Neu5Gc and Neu5Ac molecules [32]. The electrochemical detection results show that with the extension of neuraminidase hydrolysis time (Figure 4(b2)), the Neu5Gc and Neu5Ac molecules carried on the cell surface will gradually decrease, and the peak currents of PbS QDoligo and CdS QDoligo will gradually decrease (Figure 4(c2)), which further explains the sensitivity and accuracy of the electrochemical method.

The ratiometric electrochemical method has the advantage of reducing the background and avoiding environmental and instrumental influences [33]. Electrochemical signal ratio between PbS QD and CdS QD has been utilized to reflect Neu5Gc and Neu5Ac expression on the cell surface. As shown in Figure 4(d1), the electrochemical signal ratios of PbS QDoligo to CdS QDoligo decrease with the prolongation of feeding time and reach the plateau after 48 h. The results are in well agreement with those obtained for the metabolism curve of Neu5Ac and Neu5Gc (Figures 4(c1) and 4(d1)). Since neuraminidase has no selectivity for the hydrolysis of Neu5Gc and Neu5Ac glycosidic bonds, the electrochemical signal ratio of PbS QDoligo to CdS QDoligo hardly changes with the extension of neuraminidase hydrolysis time (Figure 4(d2)). These results well signify that the established ratiometric electrochemical biosensor can be applied to monitor the metabolism of Neu5Ac in the CHO cell.

### 2.5. Evaluation of Tumor Development through Ratiometric Electrochemical Method

The degree of sialylation on cell surface is related to the metastasis, invasion, and growth of cancer cells [34, 35]. The appearance of Neu5Gc on cell surface is an important event in carcinogenesis. The high expression of Neu5Gc on cell surface predicts the deterioration of cancer [36], and it is related to tumor invasion, metastasis, and grade [37]. Therefore, the detection of Neu5Gc content can not only predict tumor disease, but also give the information of tumor grade, so as to provide a reliable basis for guiding tumor medication. Bladder cancer is the most common malignancy in the urinary system, with a five-year survival rate of 75%. Most of the patients were diagnosed as nonmuscle invasive bladder cancer, 30% of which developed as muscle invasive tumor [38] and further developed into metastatic malignant tumor [16]. Therefore, the development of bladder cancer diagnosis method has become urgent. In our study, five bladder cancer cells at different stages include human normal bladder epithelial cell (HCV29) [39], grade II cancer cell (5637), muscle invasive grade III cancer cell (HT1376) [40], and transitional bladder cancer cell (J82 and T24) [39].

**Figure 5:**
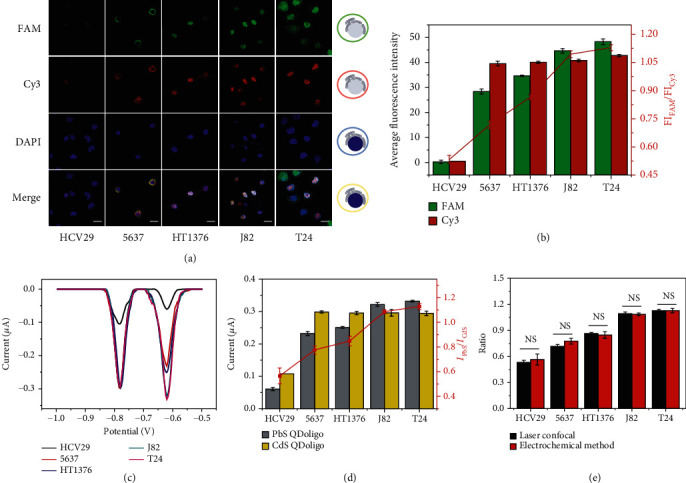
The evaluation of bladder cancer cells at different developmental stages through ratiometric electrochemical method. (a) Laser confocal images of bladder cancer cells at different developmental stages. Scale bar: 25 *μ*m. (b) Average fluorescence intensity of FAM and Cy3 obtained from fluorescence confocal microscopy by Zen software and the ratio between FAM and Cy3 fluorescence intensity for bladder cancer cells at different developmental stages. (c) Anodic stripping voltammogram for the detection of Neu5Gc and Neu5Ac on bladder cancer cells at different developmental stages. (d) Electrochemical peak current values of PbS QDoligo and CdS QDoligo and the ratio between PbS QDoligo and CdS QDoligo current values for bladder cancer cells at different developmental stages. (e) Comparison of the ratios of Neu5Gc to Neu5Ac on cell surface detected by anodic stripping voltammetry and laser confocal imaging. NS (no significance): *p* > 0.05.

Neu5Gc and Neu5Ac on the cell surface have also been detected by laser confocal microscopy, and the corresponding results have been given in Figure 5(a). After specifically recognizing Neu5Gc by FAM/DNA/Ab, Cy3/DNA/SNA will subsequently bind with Neu5Ac on cell surface. Except for HCV29, other cells show green fluorescence, suggesting the appearance of Neu5Gc, a biomarker of carcinogenesis. Moreover, mean green fluorescence intensities increase with the increasing extent of carcinogenesis (Figure 5(b)). Meanwhile, red fluorescence happens for all cells (Figure 5(a)). Except for normal bladder epithelial cell HCV29 which exhibits a low mean fluorescence intensity, all cancer cells give almost consistent high mean fluorescence intensity (Figure 5(b)). Moreover, the mean fluorescence intensity ratio of FAM and Cy3 has been calculated (Figure 5(b)). It can be found that the ratio increases with the rising grade of cancer cell.

Our established ratiometric electrochemical method has been further used to detect the content of Neu5Gc and Neu5Ac on bladder cancer cells at different stages, and the results have been given in Figure 5(c). After specific recognition of Neu5Gc by PbS/DNA/Ab, CdS/DNA/SNA further binds with Neu5Ac on cell surface. Except for HCV29 cell, other cells show the obvious electrochemical peak signals (Figure 5(c)). The current values of CdS QDoligo are almost the same for all cancer cells and signify nearly similar Neu5Ac content on the surface of these cancer cells, whereas the PbS QDoligo peak current values of different cells are arranged in descending order as follows: J82 ~ T24 > HT1376>5637 > HCV29. These results are in accordance with those obtained through laser confocal microscopy (Figures 5(a) and 5(b)). The peak current ratio of PbS QDoligo and CdS QDoligo has been further calculated, and the result has been given in Figure 5(d). The ratios increase with the increased grade of cancer cell. Furthermore, there is no statistical difference between electrochemical peak current ratio and mean fluorescence intensity ratio (Figure 5(e)). Therefore, our established electrochemical method has good accuracy and can be used to differentiate the development stage of bladder cancer.

Moreover, the bladder cancer cells at different stages of development have been added into artificial urine and further analyzed by using the established electrochemical method. As shown in Figure S4, the higher grade of bladder cancer cell, the higher electrochemical peak current of PbS QDoligo and peak current ratio between PbS QDoligo and CdS QDoligo. It well signifies that our method can distinguish bladder cancer cells from different stages of development under artificial urine environment. With the good anti-interference ability, the developed electrochemical method has potential for liquid biopsy to identify the development stage of bladder cancer with urine as sample source.

## 3. Discussion

Since the occurrence and development of tumors are very complex and even tumor cell from the same clone has the different development directions, these pose a challenge to the treatment of tumors. Therefore, the detection of staging of tumors is helpful to the layered treatment of tumors [41]. Currently, tumor development can be mainly evaluated by tissue biopsy, liquid biopsy, and radioactive imaging. The sensitivity of radioactive imaging is low, so it is impossible to detect early tumors. Tissue biopsy is limited by sample amounts. On contrary, liquid biopsy owns extensive sample sources including blood, saliva, urine, peritoneal fluid, and cerebrospinal fluid [42]. The study has shown that liquid biopsy and tissue biopsy can provide the consistent information [43]. During the development and progression of bladder cancer, the bladder cancer cells fall from the urothelium to the urine [44]. Therefore, by determining the development stage of bladder cancer cells in urine, the staging of bladder cancer can be approximately determined.

Previous studies have revealed that there is different distribution of sialic acids on the surface of tumor cell membrane, and the ratio of two kinds of sialic acids Neu5Gc and Neu5Ac is closely related to the occurrence and development of tumor. Many methods have been developed for the detection of sialic acids on the cell membrane surface (Table S2). For example, inductively coupled plasma mass spectrometry has been utilized to detect sialic acids on the surface of different cell membrane [45]. Quartz crystal microbalance method can sensitively detect sialic acid on the surface of red blood cells by using gold nanoparticles for signal amplification [46]. The surface enhanced Raman method can detect the expression of sialic acid on the surface of single HeLa cells with the usage of silver nanoparticles functionalized with 4-mercaptophenylboric acid and 4-mercaptobenzenitrile to reduce biological interference [47]; UV-Vis method can in situ detect sialic acid by constructing enzyme nanoreactor on the cell surface which can catalyze the UV color reaction [48]. Fluorescent nanorods were self-assembled by *π*-*π* stacking and hydrophobic effect using 4-(4-(pyren-1-yl) butyramido) phenylboronic acid, and two-photon imaging of sialic acid on the surface of living cells was achieved [49]. Photoelectrochemical biosensing platform modified by Ag_2_S/AuNP composites on the ITO electrode could nondestructively detect sialic acid on the surface of MCF-7 cells [50]. Electrochemical cytosensor analyzed sialic acid on the cell surface by boric acid polythiophene with the detection limit of 10 cells/mL [51]. The above various methods recognize sialic acids by boric acid, so it is impossible to detect the two kinds of sialic acids (Neu5Gc and Neu5Ac) on the cell membrane surface. In this work, a cellular interface supported CRISPR/Cas trans-cleavage strategy has been proposed through the cleverly fixing of quantum dots modified DNA strand on the surface of tumor cells by antibody or lectin, which is further combined with electrochemical technology to realize the simultaneous detection of Neu5Gc and Neu5Ac on the cell surface. Moreover, the activated Cas12a is designed to indiscriminately cleave DNA strand modified on the cell surface, resulting in the release of two kinds of PbS QDoligo and CdS QDoligo, which can be simultaneously analyzed by anodic stripping voltammetry after dissolving in nitric acid. CRISPR/Cas technology is an ultrasensitive detection method [52], which does not need time-consuming nucleic acid amplification steps and complex experimental operations such as ELISA and immune histochemistry. CRISPR/Cas technology is combined with low-cost and simple electrochemical technology [53] to realize the separate detection of two sialic acids on the cell surface. Therefore, the ratio of Neu5Gc and Neu5Ac on the cell surface can be detected by the electrochemical peak current ratio of PbS QDoligo and CdS QDoligo. The change of the ratio of two sialic acids on the cell surface reflects the occurrence and development of tumor cells. The ratiometric electrochemical method has been further developed for kinetic study of expression and hydrolysis of the sialic acids Neu5Gc and Neu5Ac on cell surface as well as evaluating bladder cancer cells at different development stages. Furthermore, our proposed method can differentiate bladder cancer cells at different stages in complex artificial urine. Therefore, our established method has great potential to predict the development period of tumor, which has important guiding significance for studying the mechanism of tumor development and drug therapy.

## 4. Materials and Methods

### 4.1. Materials and Reagents

N-acetylneuraminic acid and 4-(N-maleimidomethyl)-cyclohexane-1-carboxylic acid 3-sulfo-N-hydroxysuccinimide ester sodium salt (sulfo-SMCC) were purchased from Aladdin Co., Ltd. (Shanghai, China). Amino group-modified magnetic beads (MB) were bought from Nanjing Rui Bessie Co., Ltd (Nanjing, China). N-hydroxysuccinimide (NHS), N-(3-dimethylaminopropyl)-N'-ethylcarbodiimide hydrochloride (EDC), tris(2-carboxyethyl)-phosphine hydrochloride (TCEP), and neuraminidase were purchased from Sigma (Shanghai, China). Artificial urine, DNA, and RNA (Table S1) were obtained from Shanghai Sangon Biotechnology Co., Ltd. (Shanghai, China). The carboxylated PbS and CdS quantum dots were purchased from Shanghai Xingzi New Materials Co., Ltd. (Shanghai, China). Cas12a and anti-Neu5Gc antibody were obtained from New England Biolabs (Beijing, China). *Sambucus nigra* agglutinin (SNA) was bought from Vector Laboratories (Shanghai, China). F12k medium, RPMI-1640 medium, fetal bovine serum (FBS), and trypsin were purchased from Gibco Co., Ltd. (Beijing, China). HCV29 cell was donated by Professor Guan Feng from Northwestern University. 5637, J82, and T24 cells were donated by Professor Yang from Shanghai Tenth Hospital. CHO-K1 and HT1376 cells were purchased from the American Type Culture Collection (Shanghai, China). All experimental water was obtained by Milli-Q purification system (impedance >18 M*Ω* cm). All other chemicals were of analytical reagent grade.

### 4.2. Preparation of Neu5Gc Antibody or SNA-Labeled Single-Stranded DNA

40 *μ*g/mL SNA and 1 : 400 Neu5Gc antibody separately reacted with 1 mM sulfo-SMCC at 30°C for 2 h, followed by the centrifugation with 30 KD ultrafiltration tube at 12000 rpm to remove the unreacted sulfo-SMCC. At the same time, 2.5 *μ*M thiol group modified DNA strand (target strand, TS) was reacted with 2.5 mM TCEP solution at 37°C for 1 h. Subsequently, the activated protein solution was mixed with the reduced DNA solution at 30°C for 2 h, followed by centrifugation at 12000 rpm with a 30-KD ultrafiltration tube for 10 min to remove the unreacted DNA strand, to give Neu5Gc antibody-labeled single-stranded DNA (DNA/Ab) or SNA-labeled single-stranded DNA (DNA/SNA).

### 4.3. Preparation of Quantum Dots (QD) Labeled DNA/Ab or DNA/SNA

The 0.25 mg/mL PbS and CdS QD solution were separately mixed with the solution composed of 0.05 M NHS and 0.25 M EDC for 15 minutes at room temperature to activate carboxyl groups on the surface of QD. Subsequently, DNA/Ab was mixed with the PbS QD solution, and DNA/SNA was mixed with CdS QD solution. Both mixtures were separately reacted at 37°C for 3 h, followed by centrifugation with 30-KD ultrafiltration tube at 12000 rpm for 10 minutes, to give PbS QD-labeled DNA/Ab (PbS/DNA/Ab) or CdS QD-labeled DNA/SNA (CdS/DNA/SNA).

### 4.4. Interface Supported CRISPR/Cas Trans-Cleavage for Dual Signal Output

1 mg/mL MB and 0.5 mM sulfo-SMCC were mixed at 30°C for 2 h. After magnetic separation, 2.5 *μ*M TS, which was premixed with 2.5 mM TCEP solution at 37°C for 1 h, was further added and reacted for 2 h. Then, reaction product was mixed with the solution containing 0.25 mg/mL PbS QD and 0.25 mg/mL CdS QD, which are premixed with 0.05 M NHS and 0.25 M EDC, at 37°C for 3 h. After magnetic separation, MB@TS/PbS/CdS can be obtained. Subsequently, the mixing solution containing 200 nM Cas12a and 200 nM crRNA was added and reacted at 37°C for 30 min. After magnetic separation, the supernatant was dissolved by adding 0.1 M nitric acid. Then, anodic stripping voltammetry was conducted to detect the current of PbS QD and CdS QD, through electrodeposition at the potential of -1.2 V for 480 s. After that, differential pulse voltammetry (DPV) was performed from -1.0 V to -0.5 V with the amplitude of 50 mV. Electrochemical measurements were performed by CHI-660C electrochemical workstation with a three-electrode system. The graphite electrode deposited with a mercury film was used as the working electrode, while a platinum wire electrode and a saturated calomel electrode were separately used as auxiliary electrode and reference electrode.

### 4.5. Imaging Analysis of Neu5Gc and Neu5Ac Based on Cellular Interface Supported CRISPR/Cas Trans-Cleavage

1: 200 Neu5Gc antibody and 80 *μ*g/mL SNA reacted with 1 mM sulfo-SMCC at 30°C for 2 h, respectively. Free sulfo-SMCC was removed by centrifugation at 12000 rpm for 10 min using a 30-KD ultrafiltration tube. At the same time, 5 *μ*M FAM fluorescent group modified DNA strand with thiol group and 5 *μ*M Cy3 fluorescent group modified DNA strand with thiol group were mixed with 5 mM TCEP solution, respectively. After reaction at 37°C for 1 h, the activated Neu5Gc antibody and SNA were separately incubated with reduced FAM-modified DNA strand and Cy3-modified DNA strand at 30°C for 2 h. After centrifugation at 12000 rpm for 12 min in a 30-KD ultrafiltration centrifuge tube, Neu5Gc antibody bound to FAM-modified DNA strand (FAM/DNA/Ab) and SNA bound to Cy3-modified DNA strand (Cy3/DNA/SNA) were separately prepared. Subsequently, cells were orderly stained with FAM/DNA/Ab solution at 37°C for 40 min, Cy3/DNA/SNA solution at 37°C for 40 min, and 4',6-diamidino-2-phenylindole (DAPI) for 5 min. After washing, the cells were imaged by using a LSM 710 confocal laser scanning microscope (Zeiss, Germany).

### 4.6. Analysis of Neu5Gc and Neu5Ac Based on Cellular Interface Supported CRISPR/Cas Trans-Cleavage

200 *μ*L PbS/DNA/Ab was added into 10^4^ cells and incubated at 37°C. After 1 h, 200 *μ*L CdS/DNA/SNA was further added and incubated at 37°C for 1 h. Subsequently, the mixing solution containing 200 nM Cas12a and 200 nM crRNA was added and reacted at 37°C for 30 min. After centrifugation, the supernatant solution was detected by anodic stripping voltammetry.

### 4.7. Kinetic Study of Expression and Hydrolysis of Neu5Gc and Neu5Ac on Cell Membrane Surface

1 mM Neu5Ac was added into the cell culture medium for 1, 2, 4, 8, 12, 24, 48, 96, and 192 hours, resulting in the expression of the different amounts of Neu5Ac and Neu5Gc on the cell surface. In addition, 10^4^ cells were treated with 1 U neuraminidase to hydrolyze the Neu5Ac and Neu5Gc on the cell surface, resulting in the reduced amount of Neu5Ac and Neu5Gc on the cell surface. The amounts of Neu5Ac and Neu5Gc on the surface of the cells were detected by the constructed electrochemical method.

### 4.8. Evaluation of Tumor Development

The tumor development was evaluated by the ratio of Neu5Gc to Neu5Ac. The Neu5Gc and Neu5Ac contents of bladder cancer cells at different developmental stages were detected by electrochemistry and confocal laser scanning microscope. 10^4^ HCV29, 5637, HT1376, J82, and T24 cells were successively incubated with 200 *μ*L PbS/DNA/Ab and 200 *μ*L CdS/DNA/SNA at 37°C for 1 hour. Subsequently, 200 *μ*L solution containing 200 nM Cas12a and 200 nM crRNA was added and reacted at 37°C for 30 min. After centrifugation, the current signals of PbS and CdS QDoligo in the supernatant were detected by electrochemical method. Meanwhile, FAM/DNA/Ab and Cy3/DNA/SNA was separately used to stain bladder cancer cells such as HCV29, 5637, HT1376, J82, and T24 for 40 minutes. Then, the confocal laser scanning microscope was used for imaging, and the average fluorescence intensity of FAM and Cy3 was obtained by Zen software.

## Data Availability

All data are available within the article or supplementary information file(s). All other data supporting the findings of this study are available from the corresponding authors upon reasonable request.

## Supplementary Materials

Supplementary Materials Figure S1: Electrochemical detection of free quantum dots (QD). Figure S2: Optimization of time for dissolution of free QD by acid. Figure S3: Confocal imaging of different numbers of CHO cells. Figure S4: Ratiometric electrochemical detection of bladder cancer cells at different developmental stages in artificial urine. Table S1: Sequences of nucleic acids used in the study. Table S2: Comparison of different methods for the detection of sialic acids on cellular membrane surface.
