# Supplementary material for: Electrochemical Evaluation of Tumor Development via Cellular Interface Supported CRISPR/Cas Trans-Cleavage
Source: Research (Wash D C). 2022 Apr 6;2022:9826484. doi: 10.34133/2022/9826484 (PMC9011167; doi:10.34133/2022/9826484)
Supplement: Supplementary Materials — Figure S1: Electrochemical detection of free quantum dots (QD). Figure S2: Optimization of time for dissolution of free QD by acid. Figure S3: Confocal imaging of different numbers of CHO cells. Figure S4: Ratiometric electrochemical detection of bladder cancer cells at different developmental stages in artificial urine. Table S1: Sequences of nucleic acids used in the study. Table S2: Comparison of different methods for the detection of sialic acids on cellular membrane surface. [file 9826484.f1.docx]

**Supplementary Materials**

**Electrochemical Evaluation of Tumor Development via Cellular Interface Supported CRISPR/Cas Trans-Cleavage**

Liangfen Cheng^1^, Fuhan Yang^2^, Longfei Tang^1^ Lelin Qian^1^, Xu Chen^1^, Feng Guan^3^, Juan Zhang^1^*, Genxi Li^1,4^*

*^1^Center for Molecular Recognition and Biosensing, School of Life Sciences, Shanghai University, Shanghai 200444, P. R. China.*

*^2^Department of Urology, Shanghai Tenth People's Hospital, School of Medicine, Tongji University, Shanghai 200092, P. R. China.*

*^3^College of Life Science, Northwest University, Xi’an 710127, P. R. China.*

*^4^State Key Laboratory of Pharmaceutical Biotechnology and Collaborative Innovation Center of Chemistry for Life Sciences, Department of Biochemistry, Nanjing University, Nanjing 210093, P. R. China*

Correspondence should be addressed to Genxi Li; [genxililab@nju.edu.cn](mailto:genxililab@nju.edu.cn) and Juan Zhang; [juanzhang@shu.edu.cn](mailto:juanzhang@shu.edu.cn)

**1 Sequences of nucleic acids**

Table S1 Sequences of nucleic acids used in the study.

| Name | Sequences |
| --- | --- |
| crRNA | UAA UUU CUA CUA AGU GUA GAU CCU CGG CGU GCA GUC UCU UC |
| Target strand (TS) | NH_2_-TTT TTT TTT TCC G AT GGA AGA GAC TGC ACG CCG AGG TAA AGG GTC AGT TTT TTT TTT-SH |
| FAM modified DNA strand (FAM/DNA) | FAM-TTT TTT TTT TCC G AT GGA AGA GAC TGC ACG CCG AGG TAA AGG GTC AGT TTT TTT TTT-SH |
| Cy3 modified DNA strand (Cy3/DNA) | Cy3-TTT TTT TTT TCC G AT GGA AGA GAC TGC ACG CCG AGG TAA AGG GTC AGT TTT TTT TTT-SH |

**2 Electrochemical detection of free quantum dots (QD)**

**
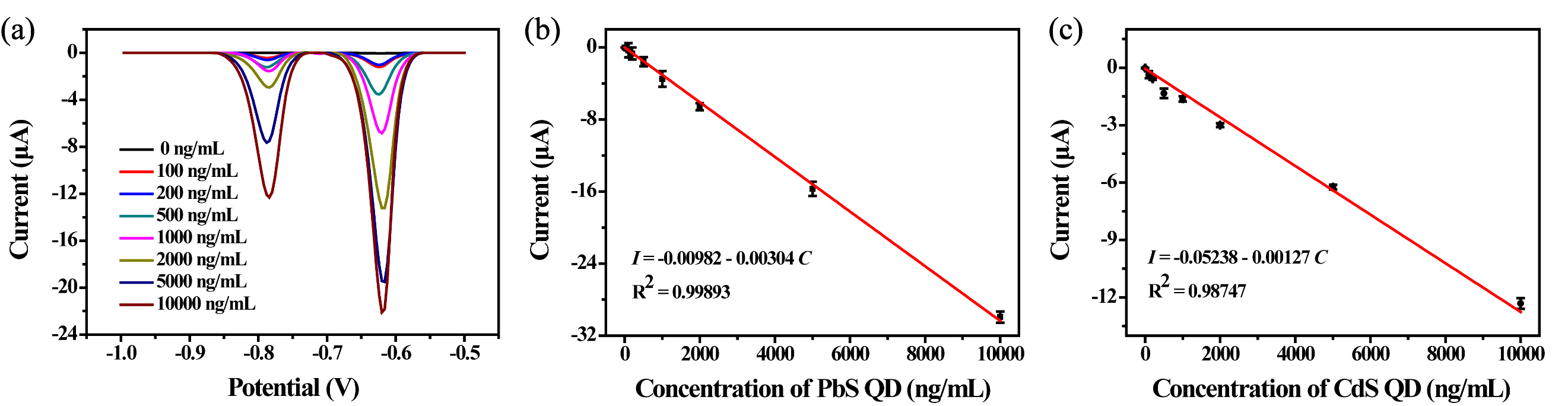
**

Figure S1 (a) Differential pulse voltammogram obtained by mixing PbS QD and CdS QD at different concentrations with molar ratio of 1:1. Linear relationships between current values and the concentrations of (b) PbS QD or (c) CdS QD: 0, 100, 200, 500, 1000, 2000, 5000, 10000 ng/mL.

**3 Optimization of experimental conditions**


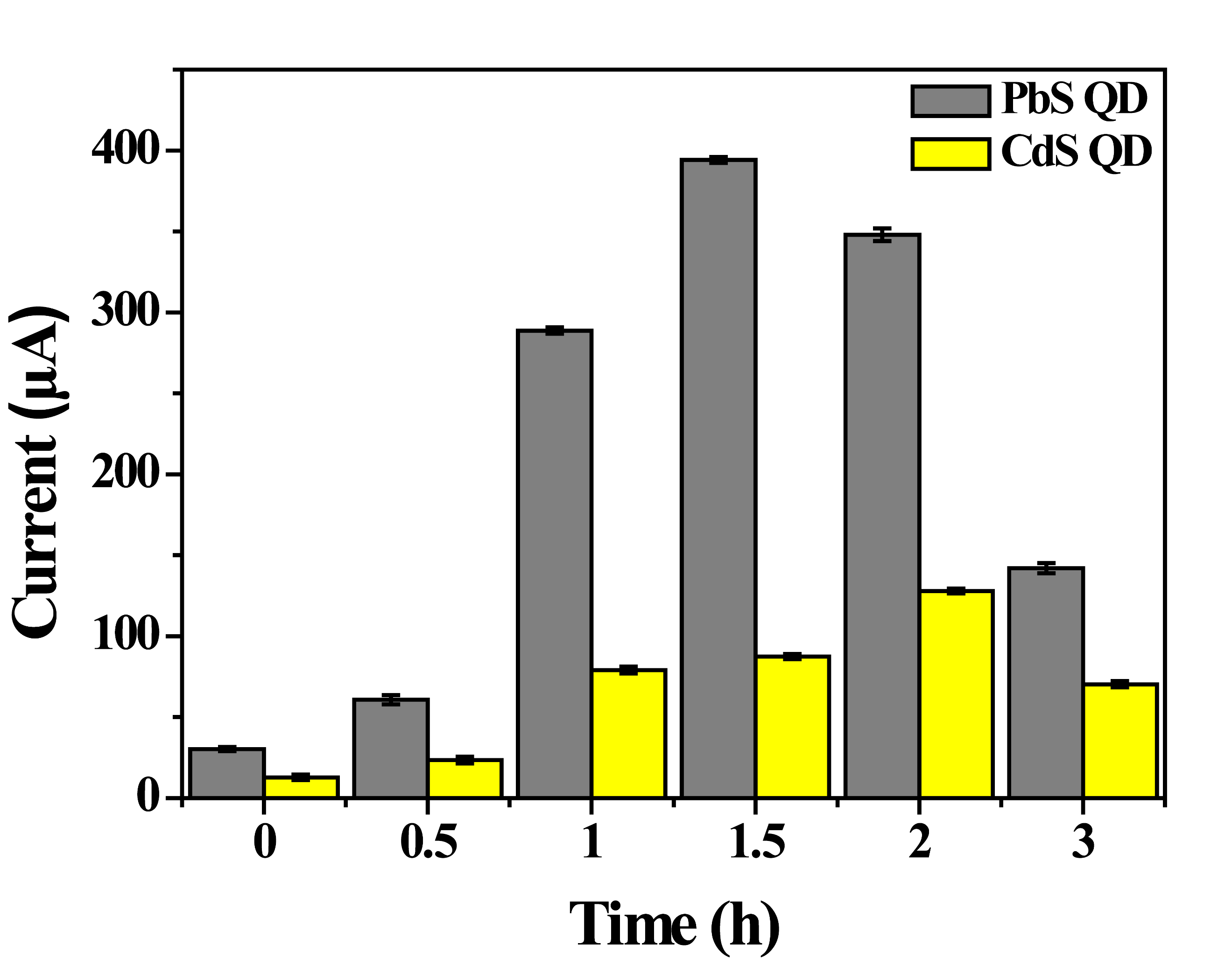


Figure S2 Optimization of time for dissolution of free QD by nitric acid and the optimal time is 2 h.

**
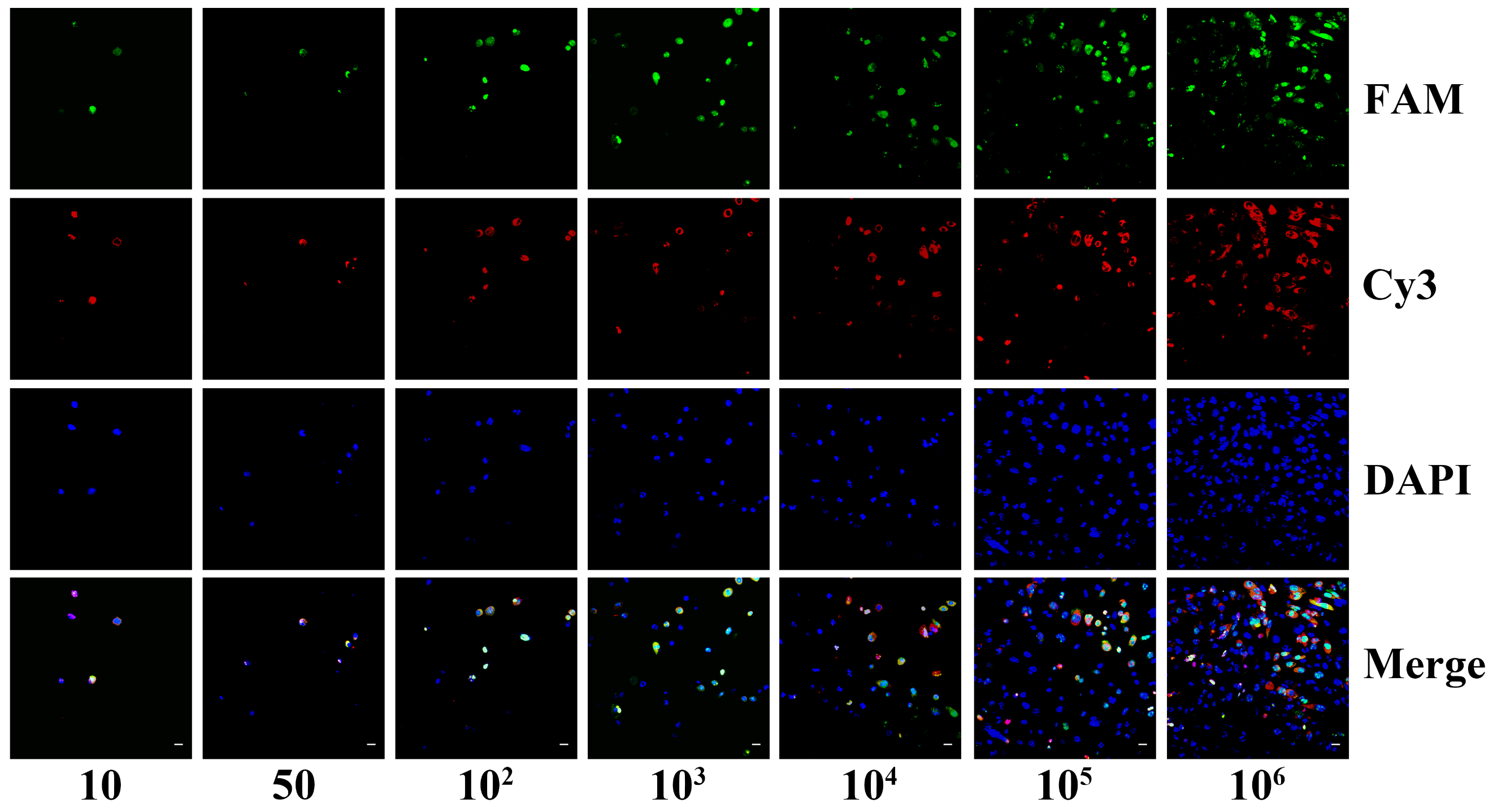
4 Confocal imaging of different numbers of CHO cells**

Figure S3 Confocal images of different numbers of CHO cells which were incubated with FAM/DNA/Ab and Cy3/DNA/SNA. Scar bar: 25 μm

**5 Electrochemical detection of bladder cancer cells at different developmental stages in artificial urine**


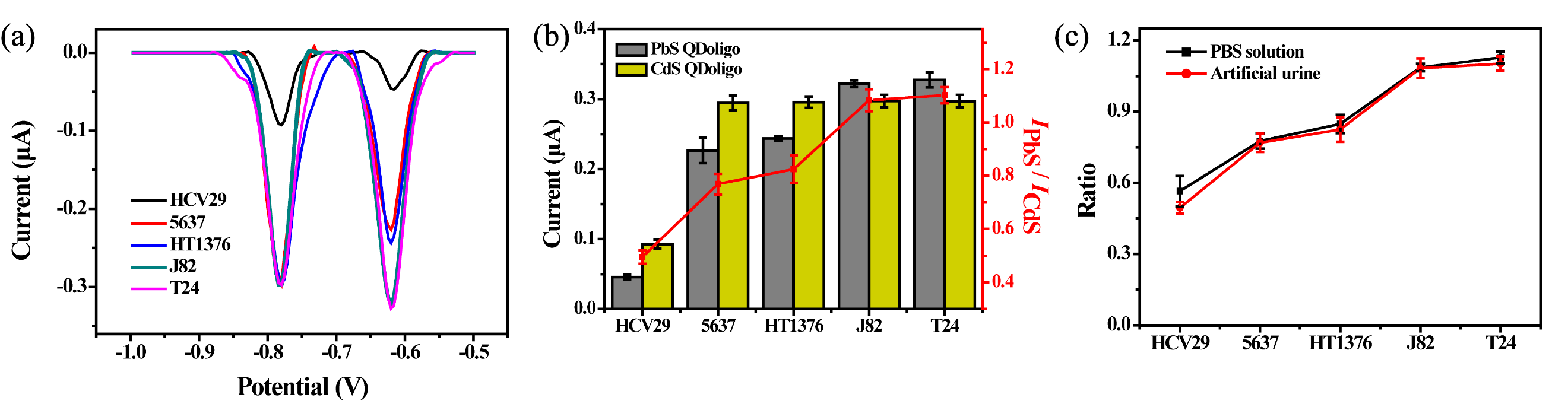


Figure S4 Ratiometric electrochemical detection of bladder cancer cells at different developmental stages in artificial urine (a) Anodic stripping voltammogram for the detection of Neu5Gc and Neu5Ac on bladder cancer cells at different developmental stages in artificial urine. (b) Electrochemical peak current values of PbS QDoligo and CdS QDoligo for bladder cancer cells at different developmental stages in artificial urine and peak current ratio between PbS QDoligo and CdS QDoligo. (c) The ratio of Neu5Gc to Neu5Ac in bladder cancer cells at different developmental stages in PBS solution and artificial urine.

**6 Comparison of different methods for the detection of sialic acids on cellular membrane surface**

Table S2 Comparison of different methods for the detection of sialic acids on cellular membrane surface

| Detection system | Detection target | Advantages | Component | references |
| --- | --- | --- | --- | --- |
| Inductively coupled plasma mass spectrometry | Sialic acid on cell surface | Sensitive and selective (detection limit: 640 cells/mL) | Streptavidin conjugated AuNPs labelled on biotin- aminobenzeneboronic acid | [45] |
| Quartz crystal microbalance | Sialic acid on cell surface | In situ and high sensitivity (detection limit: 1100 cells/mL) | Aminobenzeneboronic acid -functionalized gold nanoparticles | [46] |
| Surface enhanced Raman scattering | Sialic acid on cell surface | Whole surface accessibility, no background interference and high sensitivity | [Silver nanoparticles](https://www.sciencedirect.com/topics/chemistry/silver-nanoparticle) functionalized with 4-mercaptophenylboric acid and 4-mercaptobenzenitrile | [47] |
| UV–vis | Sialic acid on cell surface | *In situ*, excellent biocompatibility | Poly(styrene-co-maleic anhydride-acrylic acid-concanavalin A) | [48] |
| Fluorescence imaging | Sialic acid on cell surface | *In situ,* simple and effective | 4-(4-(pyren-1-yl)butyramido)phenylboronic acid nanorods | [49] |
| Photoelectrochemical | Sialic acid on cell surface | Nondestructive analysis of living cells (detection limit: 100 cells/mL) | Ag_2_S/AuNP composites | [49] |
| Electrochemical | Sialic acid on cell surface | Low-cost, highly sensitive and selective cytosensor (detection limit: 10 cells/mL) | Boronic acid-functionalized polythiophene | [50] |
| Electrochemical | Neu5Gc, Neu5Ac on cell surface | Simple, sensitive (detection limit: 1.12 cells/mL) | CRISPR/Cas system | This work |
